# Sulforaphane Inhibits Invasion via Activating ERK1/2 Signaling in Human Glioblastoma U87MG and U373MG Cells

**DOI:** 10.1371/journal.pone.0090520

**Published:** 2014-02-28

**Authors:** Chunliu Li, Yan Zhou, Xiaohui Peng, Lianlian Du, Hua Tian, Gaoxiang Yang, Jing Niu, Wei Wu

**Affiliations:** 1 Department of Epidemiology and Health Statistics, School of Public Health, Capital Medical University, Beijing, China; 2 Department of Biochemistry and Molecular Biology, School of Basic Medical Sciences, Capital Medical University, Beijing, China; 3 Beijing Municipal Key Laboratory of Clinical Epidemiology, School of Public Health, Capital Medical University, Beijing, China; Seoul National University, Republic of Korea

## Abstract

**Background:**

Glioblastoma has highly invasive potential, which might result in poor prognosis and therapeutic failure. Hence, the key we study is to find effective therapies to repress migration and invasion. Sulforaphane (SFN) was demonstrated to inhibit cell growth in a variety of tumors. Here, we will further investigate whether SFN inhibits migration and invasion and find the possible mechanisms in human glioblastoma U87MG and U373MG cells.

**Methods:**

First, the optimal time and dose of SFN for migration and invasion study were determined via cell viability and cell morphological assay. Further, scratch assay and transwell invasion assay were employed to investigate the effect of SFN on migration and invasion. Meanwhile, Western blots were used to detect the molecular linkage among invasion related proteins phosphorylated ERK1/2, matrix metalloproteinase-2 (MMP-2) and CD44v6. Furthermore, Gelatin zymography was performed to detect the inhibition of MMP-2 activation. In addition, ERK1/2 blocker PD98059 (25 µM) was integrated to find the link between activated ERK1/2 and invasion, MMP-2 and CD44v6.

**Results:**

The results showed that SFN (20 µM) remarkably reduced the formation of cell pseudopodia, indicating that SFN might inhibit cell motility. As expected, scratch assay and transwell invasion assay showed that SFN inhibited glioblastoma cell migration and invasion. Western blot and Gelatin zymography showed that SFN phosphorylated ERK1/2 in a sustained way, which contributed to the downregulated MMP-2 expression and activity, and the upregulated CD44v6 expression. These molecular interactions resulted in the inhibition of cell invasion.

**Conclusions:**

SFN inhibited migration and invasion processes. Furthermore, SFN inhibited invasion via activating ERK1/2 in a sustained way. The accumulated ERK1/2 activation downregulated MMP-2 expression and decreased its activity and upregulated CD44v6. SFN might be a potential therapeutic agent by activating ERK1/2 signaling against human glioblastoma.

## Introduction

Glioblastoma is one of the most common and fatal tumors [1]. It is composed of poorly differentiated neoplastic astrocytes with highly invasive potential. The poor prognosis of glioblastoma primarily results from the severe invasiveness. The commonly used strategies for treatment include surgery, radiation, and chemotherapy. Chemotherapy has been shown to modestly increase survival in patients who failed in surgery and radiotherapy [2]. Implantation of carmustine polymer into the resection cavity before radiotherapy has been confirmed to improve the median survival compared with radiotherapy alone [3]. The first line drug for chemotherapy is the alkylating agent temozolomide (TMZ). Unfortunately, glioblastoma cells often generated resistance to TMZ treatment [4]. More, in nearly 20% of patients treated with TMZ, significant clinical toxicity was observed regularly [5]. Due to the side effects, chemotherapy with TMZ only achieved limited efficiency. Therefore, there will be a substantial need for effective and safe chemotherapeutic agents.

It was reported that dietary intake of cruciferous vegetables is negatively related to cancer risk in epidemiological studies [6]. The protective effect is demonstrated to derive from the isothiocyanate activity in such vegetables. Sulforaphane (SFN), 1-isothiocyanato-4-(methylsulfinyl)-butane, is one of the most extensively characterized isothiocyanates rich in the brassica oleracea family of cruciferous vegetables with potential anti-carcinogenic properties [7]. The mechanisms of protection against the initiation of carcinogenesis by SFN include inhibiting DNA adduct formation, and reducing the risk of mutations. SFN targets cancer cells and prevents their growth by inhibiting proliferation and inducing apoptosis [8]. Recently, the effect of SFN on tumor cell migration and invasion was reported. Studies showed that SFN inhibited migration in prostate cancer, invasion in breast cancer, and inhibited migration and invasion both in human bladder cancer and oral carcinoma [9], [10], [11], [12]. However, the effect of SFN on glioblastoma migration and invasion has not been reported yet. Therefore, in this study, we examined the glioblastoma cell lines U87MG and U373MG in order to better understand the effect of SFN on the migration and invasion of the cells. In the preliminary study we found SFN inhibited migration and invasion, but the involved mechanisms are unclear.

The MEK/ERK pathway plays essential roles in cell survival, differentiation, apoptosis, proliferation, migration and invasion [13], [14], [15], [16]. Some studies reported that acutely (5–15 min stimulation with cytokine) activated ERK1/2 signaling contributed to cell proliferation, migration and invasion [17], [18], [19], [20]. But reports showed that sustained (more than 15 min stimulation with cytokine) activation of ERK1/2 led to reduction of cell proliferation, induced cell cycle arrest, differentiation and apoptosis [16], [21], [22], [23], [24]. We ascribed the converse cellular responses to the duration of ERK1/2 activation [16]. Studies showed that SFN activated ERK1/2 and induced apoptosis in human brain glioma cells and neuroblastoma cells [25], [26]. We have demonstrated that transient activation of ERK1/2 contributed to glioblastoma migration and invasion [27], [28]. Thus, here we will investigate whether SFN inhibits invasion and regulates ERK1/2 and its downstream effectors in human glioblastoma cells.

Basement membrane and extracellular matrix provide the main physical barriers against cancer cell invasion. Thus, the matrix metalloproteinases (MMPs), which might degrade basement membrane, have been proposed to be critical in the invasion processes [29]. Increased expression of MMP-2 is reported in many human tumors, including breast, ovarian, prostate tumors, and melanoma [30]. Human glioblastoma samples express elevated levels of MMP-2 compared with low grade brain tumors and normal brain tissues [31]. In particular, MMP-2 is highly expressed in glioblastoma and its expression increases with tumor progression at both the mRNA and protein levels [32]. Recent studies showed that SFN inhibited MMP-2 expression in some tumor cell lines [11], [12]. Moreover, our previous studies and others have confirmed that MMP-2 was the downstream effector of ERK1/2 [27], [28], [33], [34]. Therefore, we proposed that SFN might down-regulate MMP-2 via activating ERK1/2 signaling pathway.

Tumor migration and invasion are complicated processes involving many molecules. Adhesion molecules are known to play an important role in tumor cell migration and invasion. CD44 glycoproteins constitute one of five major families of cell adhesion molecules. Alternative splicing of CD44 mRNA in 10 of the 20 exons generates several variant CD44 isoforms. The variant isoform CD44v6 has attracted increasing interest since a study showed its participation in tumor metastasis [35]. It was reported that ERK1/2 regulation of CD44 modulates oral cancer aggressiveness [13]. Thus, we assumed that SFN might regulate CD44v6 and contribute to invasion inhibition via activating ERK1/2 pathway in human glioblastoma U87MG and U373MG cells.

The aim of this study is to evaluate the ability of SFN to suppress glioblastoma cell migration and invasion, and to reveal the underlying molecular mechanisms. This study would provide a scientific basis for clinically using SFN safely and effectively to inhibit glioblastoma.

## Materials and Methods

### Reagents

D,L-Sulforaphane (SFN) was acquired from Sigma (St Louis, MO, USA). Dimethyl sulfoxide (DMSO) was bought from AppliChem GmbH (Ottoweg4, D-64291 Darmstadt, Germany). MTS assay kit was purchased from Promega (Madison, USA). DMEM/HIGH glucose culture medium was purchased from Hyclone (Logan, Utah, USA). Fetal bovine serum (FBS) and penicillin–streptomycin were acquired from Invitrogen (Carlsbad, CA, USA). Transwell plates for invasion assay were purchased from BD Biosciences (Bedford, MA, USA). The antibodies, anti-ERK1/2, anti-phospho-ERK1/2 and anti-MMP-2 were purchased from Cell Signaling Technology, Inc (Shanghai, China). Anti-CD44v6 was purchased from Abcam (Hong Kong).

### Cell Culture

Human glioblastoma cell line (U87MG) was purchased from the Cell Resource Center, Peking Union Medical College (CRC/PUMC). U373MG was purchased from American Type Culture Collection (ATCC, USA). The two cell lines are commonly used as models of glioblastoma and present a spectrum of different genetic lesions [36]. The cells were maintained in DMEM/HIGH glucose culture medium supplemented with 10% FBS, 100 U/ml penicillin and 100 U/ml streptomycin in a standard humidified incubator containing 5% CO_2_ at 37°C. The medium was refreshed every 2 days. Cells were trypsinized by trypsin-EDTA. The cells in the logarithmic growth phase were used to conduct the experiments described as follows. All experiments were done in triplicate.

### MTS Assay

SFN was dissolved in DMSO, stored at −20°C and diluted to the desired concentration immediately before the experiments. The final amount of the DMSO did not exceed 0.1% v/v. The cell viability was determined using the CellTiter 96® AQueous One Solution Cell Proliferation Assay (MTS Assay) kit according to the manufacturer’s instructions. The cells were plated in 96-well plates at 4–6×10^3^ cells per well overnight. Then, the FBS-free media containing various doses of SFN were used. After 24 h, 20 µl of the pre-warmed MTS reagent was added to each of the wells and the 96-well plates were incubated at 37°C in a humidified incubator supplied with 5% CO_2_ for 1 h. The absorbance was recorded at 490 nm using a BioTek® microplate reader (SynergyTM HT, USA). The cell viability was calculated as the percentage of the measured absorbance of the control group treated with 0.1% DMSO v/v. Each assay was performed in triplicate, and the results were expressed as the mean (± SD).

### Morphological Observation

U87MG and U373MG cells were grown to 70%–80% confluence in 6-well culture plates. Then various doses of SFN were added to the media. Morphological changes were documented with a phase-contrast microscope at ×100 magnification (Leica, Germany) connected to a digital camera (Olympus, Japan) at different time points. At 3–5 vision fields were chosen to see the cell morphology.

### Scratch Assay

The glioma cells were grown to confluence in 6-well culture plates. Then they were scratched with 200 µl pipette tips. After the suspended cells were washed for three times, the wounded monolayers were cultured in FBS-free media with various doses of SFN for 24 h. Closure of the wounded areas was observed under a phase-contrast microscope at ×40 magnification (Leica, Germany) and quantified with the NIH Image J image processing program. The wound area was photographed at the indicated time intervals (T = 0, 24 h) with Olympus DP71 camera (Japan). These experiments were performed in triplicate.

### Invasion Assay

Cell invasion was performed using BD BioCoat Matrigel™ invasion chambers (BD Biosciences, USA) pre-coated with BD Matrigel matrix. The 24-well artificial basement membrane inserts had 8 µm pores allowing these single cells to invade. The assay insert plates were prepared by rehydrating the BD Matrigel with 300 µl pre-warmed serum-free medium at room temperature for 30 min. The remaining medium was carefully removed. The number of 1×10^5^ of control cells, SFN (optimal dose acquired by the MTS assay and morphological observation ) treated cells and SFN combined with PD98059 (25 µM) treated cells suspended in 300 µl DMEM medium without FBS were added separately onto the apical chambers. A volume of 500 µl DMEM medium with 10% FBS was added to the basal chambers. Assay plates were incubated for 24 h incubation in 37°C, 5% CO_2_ incubator. The non-invading cells on the top chamber were removed gently with a cotton swab. The invaded cells on the bottom of the chamber were fixed with 100% methanol for 20 min at −20°C, and then stained with 0.5% crystal violet solution (made in 25% methanol) at room temperature for 20 min. After the crystal violet solution was moved away, the cells were rinsed with distilled water until excess dye was removed. Twelve vision fields were selected randomly per well under a microscope and the number of cells that penetrated the membrane was counted. Images were acquired with a Leica DMIRB microscope at ×40 magnification.

### Immunoblotting

U87MG and U373MG cells were collected and lysed with lysis buffer (25 mM Tris-HCl pH 7.6, 150 mM NaCl, 1% NP-40, 0.1% SDS, 1% sodium deoxycholate and protease inhibitors, Thermo Scientific, USA). Cell lysate was centrifuged at 18,000×g for 20 min. Total protein concentrations were tested by BCA Protein Assay Kit (Invitrogen, Carlsbad, CA, USA). Equal amounts of total protein were loaded on 10% SDS-PAGE gels and run for required time depending on the molecular weight, then transferred to nitrocellulose membranes via semi-dry transfer. The membranes were blocked in 1.5% BSA in TBS-Tween 20 (TBS-T) buffer for 1 h at room temperature with gentle shaking, incubated at 4°C overnight with primary antibodies against ERK1/2, phospho-ERK1/2, MMP-2, CD44v6, and α-tubulin. After washing with TBS-T for 3×10 minutes each, the membranes were probed with the fluorescence-labeled secondary antibody (LI-COR Bioscience, Lincoln, NE, USA) for 1 h at room temperature. After three washes, the membranes were scanned in both the 700 and 800 channels using the Odyssey Infrared Imaging System (LI-COR Bioscience). The band for α-tubulin was used for equal loading and normalization. Antibodies were diluted appropriately following the protocols provided by the vendors.

### Gelatin Zymography

MMP-2 activity was measured using gelatin gel zymography. After we changed the 10% FBS DMEM medium into serum-free medium, the ERK1/2 blocker PD98059 (25 µM) or SFN (appropriate dose) was applied to treat cells, and cultured for 24 h. Medium with secreted MMP-2 protein was collected from an equal number of cells and was centrifuged at 2000 rpm for 10 min to remove cellular debris. Then we collected the supernatant and mixed it with equal amount of sample buffer for loading in the gel. The equal volumes of medium were run on 10% SDS- polyacrylamide gel containing 1 mg/ml gelatin as a protease substrate. The gel was washed in 2.5% Triton X-100 solution at room temperature with gentle agitation 2×30 minutes each, followed by incubation at 37°C for 42 h in the buffer (50 mM Tris-HCl, pH 7.5, 0.2 M NaCl, 5 mM CaCl_2_·2H_2_O, and 0.02% Brij-35, pH 7.6). After incubation, the gel was stained for 40 min with staining solution (0.5% Coomassie Brilliant Blue, 25% isopropanol, and 10% acetic acid). Then we destained the stained gel with an appropriate Coomassie R-250 destaining solution (50% methanol, 10% acetic acid). At last, we could see dark blue background against unstained regions of protease-digested gelatin.

### Statistical Analysis

Data are expressed as means ± SD. Differences were evaluated using one-way ANOVA for multiple comparisons and t tests for 2-group comparisons. All statistical analyses were performed using SPSS 18.0 software package. Every experiment was repeated at least three times. *P<0.05* was considered statistically significant.

## Results

### SFN Inhibited Cell Viability in a Dose-dependent Manner

**Figure 1:**
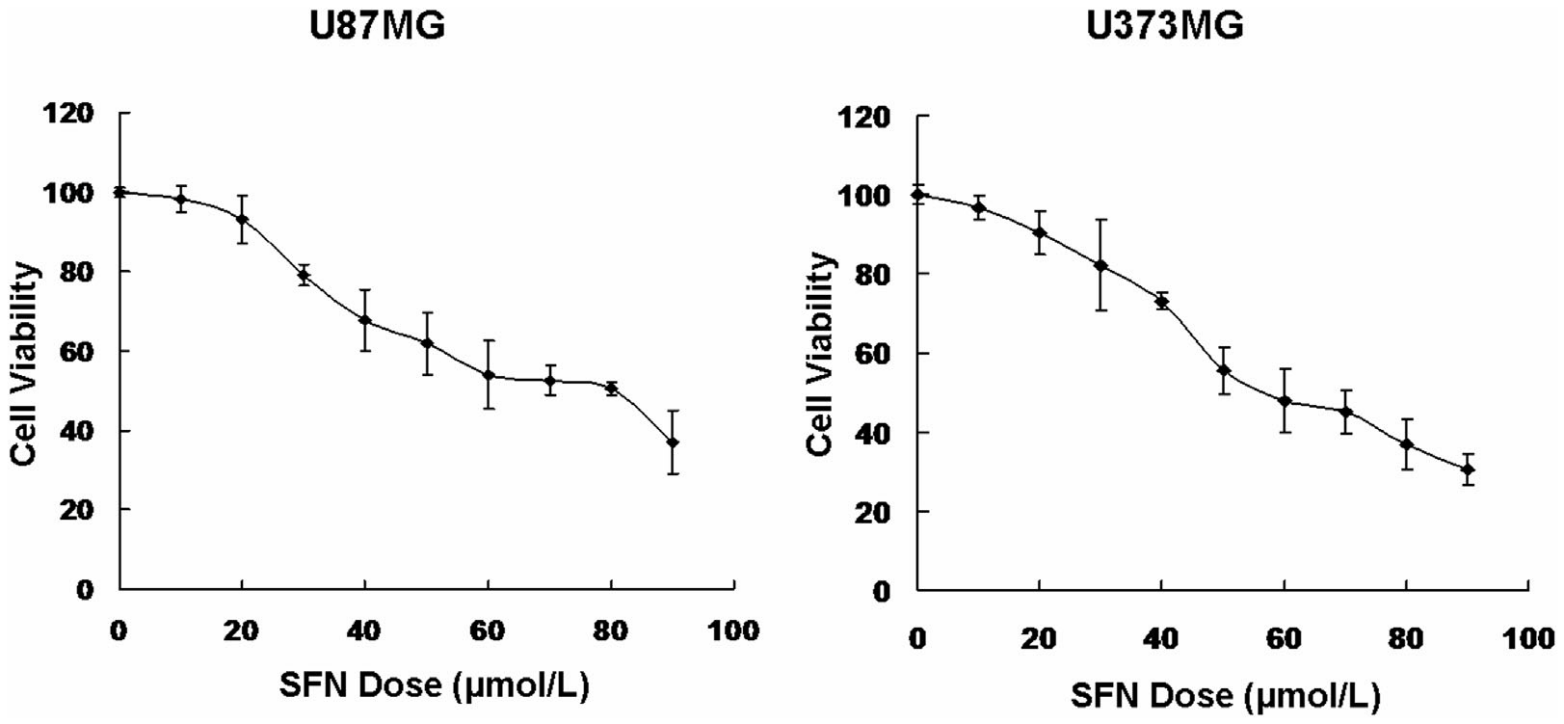
SFN inhibited cell viability. An in(0, 10, 20, 30, 40, 50, 60, 70, 80 and 90 µM) for 24 h. The viability of the SFN-treated cells was measured using the MTS assay. Results were expressed as a percentage of control, which was considered as 100%. Data were reported as mean ± SD and at least three separate experiments were performed.

SFN inhibited cell growth in a variety of tumors, thus we detected if SFN inhibited the viability of U87MG and U373MG cells. In order to obtain the dose-response curve, we treated tumor cells with the increasing doses of SFN (0, 10, 20, 30, 40, 50, 60, 70, 80, and 90 µM) for 24 h. The treatment time 24 h was determined by our previous study, which the treatment duration might cause a sustained ERK1/2 activation [27], [28]. The viability of the SFN-treated cells was then measured by MTS assay. The results showed that the cell viability was decreased after treated by SFN (more than 20 µM) for 24 h in a dose-dependent manner (Figure 1). Meanwhile, we found that SFN did not decrease cell viability significantly at the dose of 20 µM. Therefore, we will use 20 µM as the optimal dose of SFN for our further studies, especially for migration and invasion test.

### SFN Changed Cell Morphology in Dose- and Time-dependent Manners

**Figure 2:**
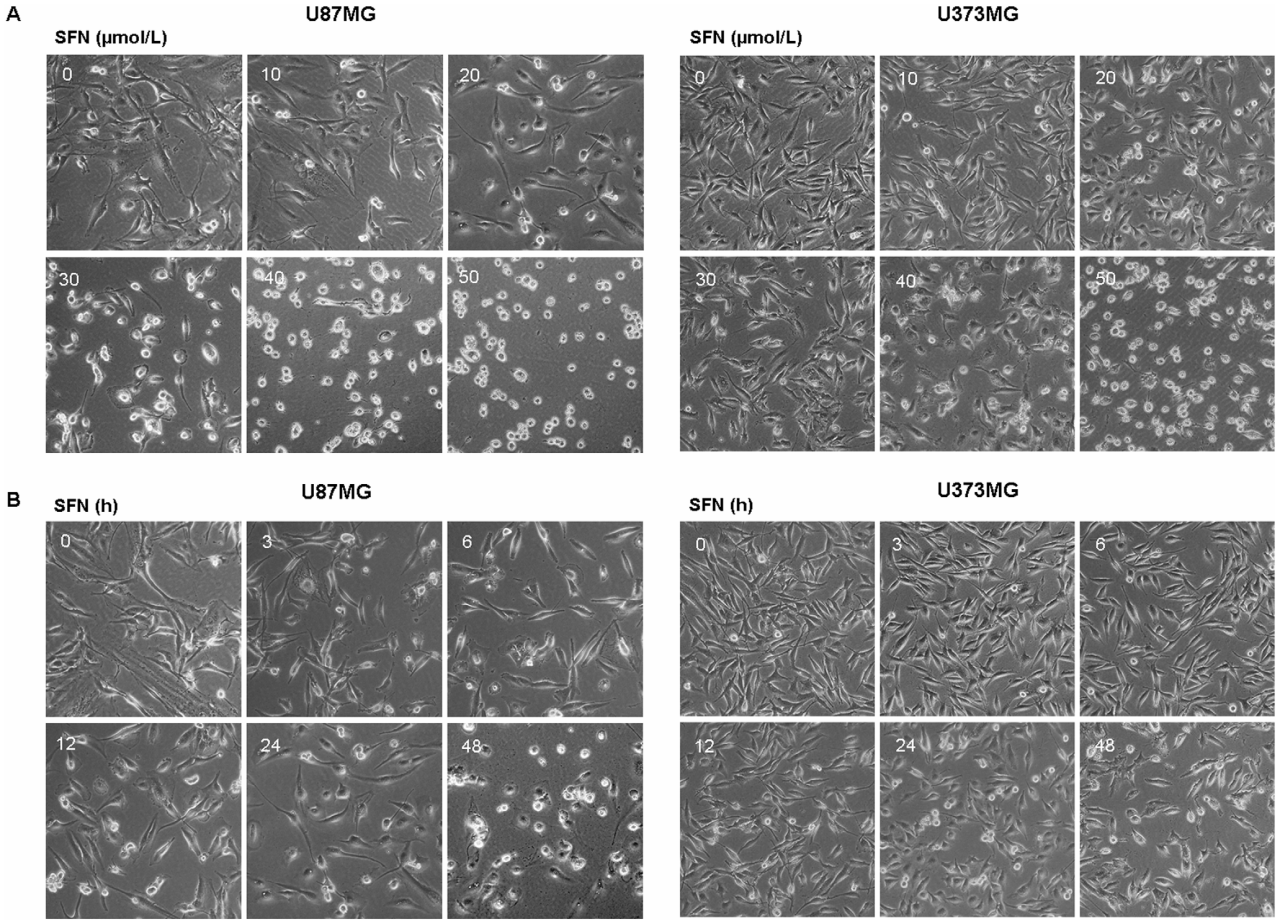
SFN changed cell morphology. (A) Cellular morphological changes in U87MG and U373MG cell lines were done in a dose-dependent manner after treated with SFN for 24 h when observed by a Leica DMIRB Microscope at ×100 magnification. (B) Cellular morphological changes in U87MG and U373MG cell lines were done in a time-dependent manner after treated with 20 µM SFN when observed by a Leica DMIRB Microscope at ×100 magnification.

Morphological observation showed that SFN-treated cells exhibited smooth surfaces with obvious reduction of pseudopodia (Figure 2A, Figure 2B). Moreover, in our experiments, cellular morphology changed in both dose- and time-dependent manners. Thus, we proposed that SFN might inhibit cell migration and invasion in U87MG and U373MG cells. Considering that there was no significant death in U87MG and U373MG cells treated with 20 µM SFN for 24 h, and simultaneously obvious morphological changes were observed, we chose 20 µM, 24 h as the optimal dose and time for further study.

### SFN Inhibited Migration in U87MG and U373MG Cells

**Figure 3:**
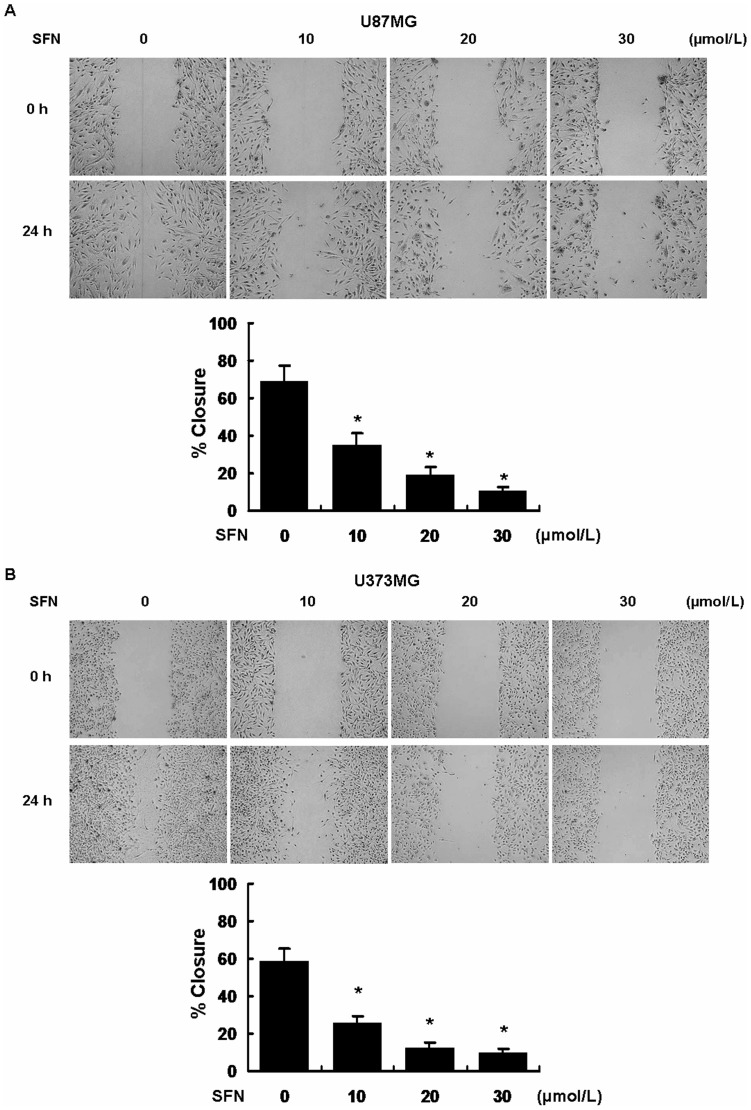
SFN inhibited migration in U87MG and U373MG cell lines. Confluent U87MG and U373MG cells were scratched and incubated at different concentrations of SFN (µM). The area covered by migrating cells was recorded by phase-contrast microscopy connected to a digital camera at time 0 and 24 h. The wound closure area was calculated by measuring the diminution of the wound bed surface upon time using Image J software. Representative pictures of three independent experiments were shown. *, indicates *P<0.05* versus no SFN group.

We next performed a commonly used wound healing assay - scratch assay, to determine the influence of SFN on cell migration over a period of 24 h. Figure 3 shows that the cells infiltrated the gap after 24 h in the control group. SFN significantly decreased cell migration versus the control group in U87MG and U373MG cells.

### SFN Inhibited Invasion via a Dose-dependent Manner

**Figure 4:**
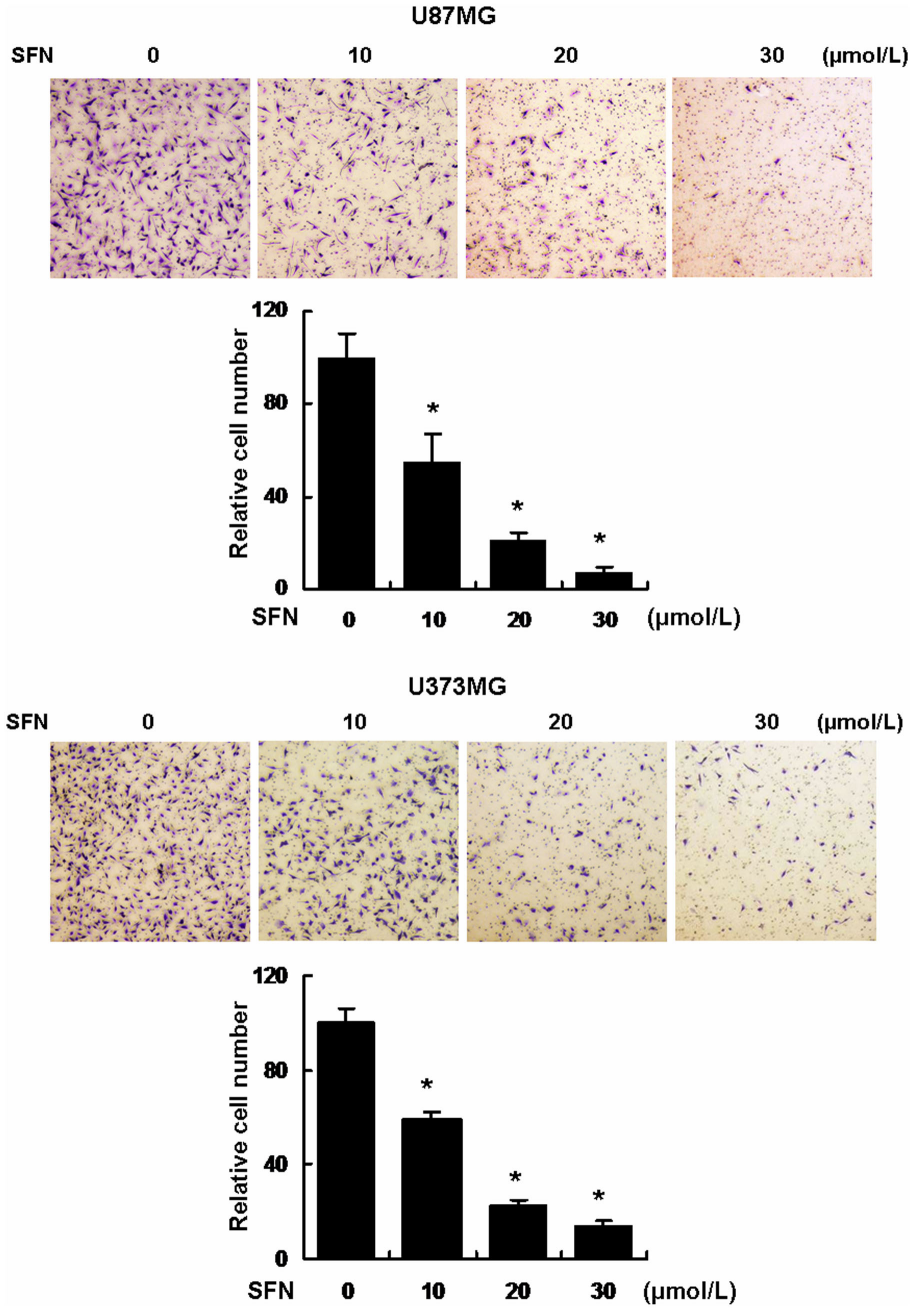
SFN inhibited invasion in a dose-dependent manner in U87MG and U373MG cell lines. Approximately 1×10^5^ cells were seeded in the 24-well plate with cell culture inserts, the cells were treated with different concentrations of SFN (µM) for 24 h to test invasion. Assays were performed as described in Materials and Methods. The results showed that SFN inhibited significantly cell invasion in a dose-dependent manner. *, indicates *P<0.05* versus no SFN group. Data were shown as means ± SD from three independent experiments.

To determine whether SFN weakens the cell invasive potential, Transwell matrigel invasion assays were conducted. The cells invading through the matrigel were counted. The results showed that the cell numbers in the following groups (10, 20, 30 µM) treated with SFN for 24 h were significantly decreased versus control cells (0 µM) following a dose-dependent manner (Figure 4). Clearly, SFN attenuated the cell invasive ability significantly.

### SFN Activated ERK1/2 in Dose- and Time-dependent Manners

**Figure 5:**
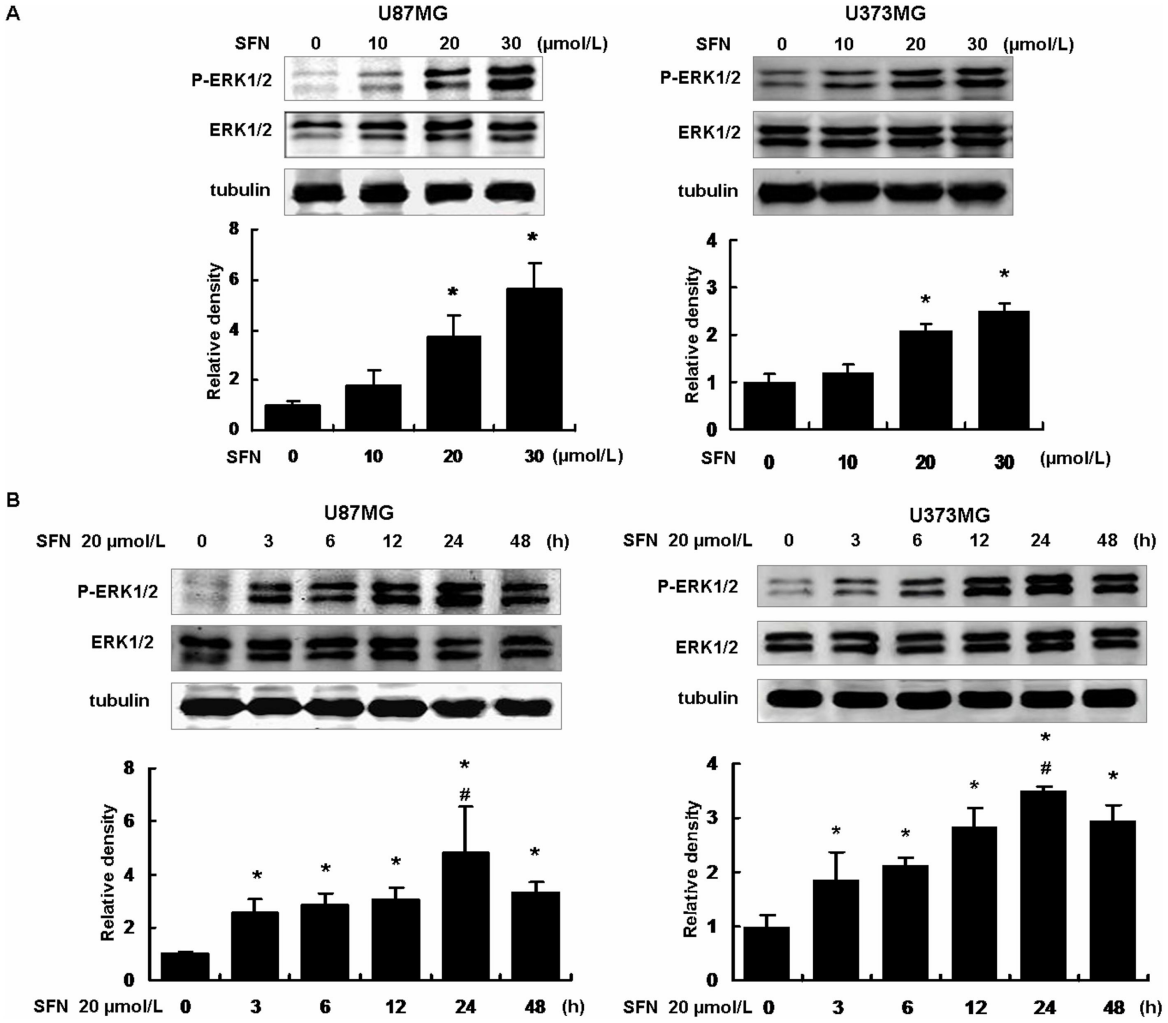
SFN phosphorylated ERK1/2 in a sustained way. (A) We have demonstrated that SFN inhibited cell migration and invasion in a dose-dependent manner. Thus, here we treated the U87MG and U373MG cells with different doses of 0, 10, 20, and 30 µM SFN for 24 h. Western blot showed that SFN phosphorylated ERK1/2 in a dose-dependent manner. At the concentration of 20 µM, ERK1/2 activation was significantly increased. (B) The cells were treated with 20 µM SFN for 0, 3, 6, 12, 24 and 48 h. Western blot showed that SFN activated ERK1/2 in a sustained way. At the time point of 24 h, ERK1/2 phosphorylation reached the peak. *, indicates *P<0.05* versus no SFN group, #, indicates *P<0.05* versus other groups. Data were shown as means ± SD from three independent experiments.

Since we have found that SFN inhibited cell migration and invasion in U87MG and U373MG cells, we tried to characterize the involved molecular mechanisms. SFN was added to the medium at the doses of 0, 10, 20 and 30 µM for 24 h. Western blot showed that SFN increased ERK1/2 phosphorylation in a dose-dependent manner. Phosphorylation of ERK1/2 was increased significantly once the cells were treated with 20 µM SFN (Figure 5A). Furthermore, once the cells were treated with 20 µM SFN at different time points (0, 3, 6, 12, 24, 48 h), the results showed that ERK1/2 phosphorylation was increased to peak when the cells were treated for 24 h (Figure 5B). These results indicated that SFN activated ERK1/2 in a time-dependent manner.

### SFN Inhibited Invasion via Sustained Activation of ERK1/2

**Figure 6:**
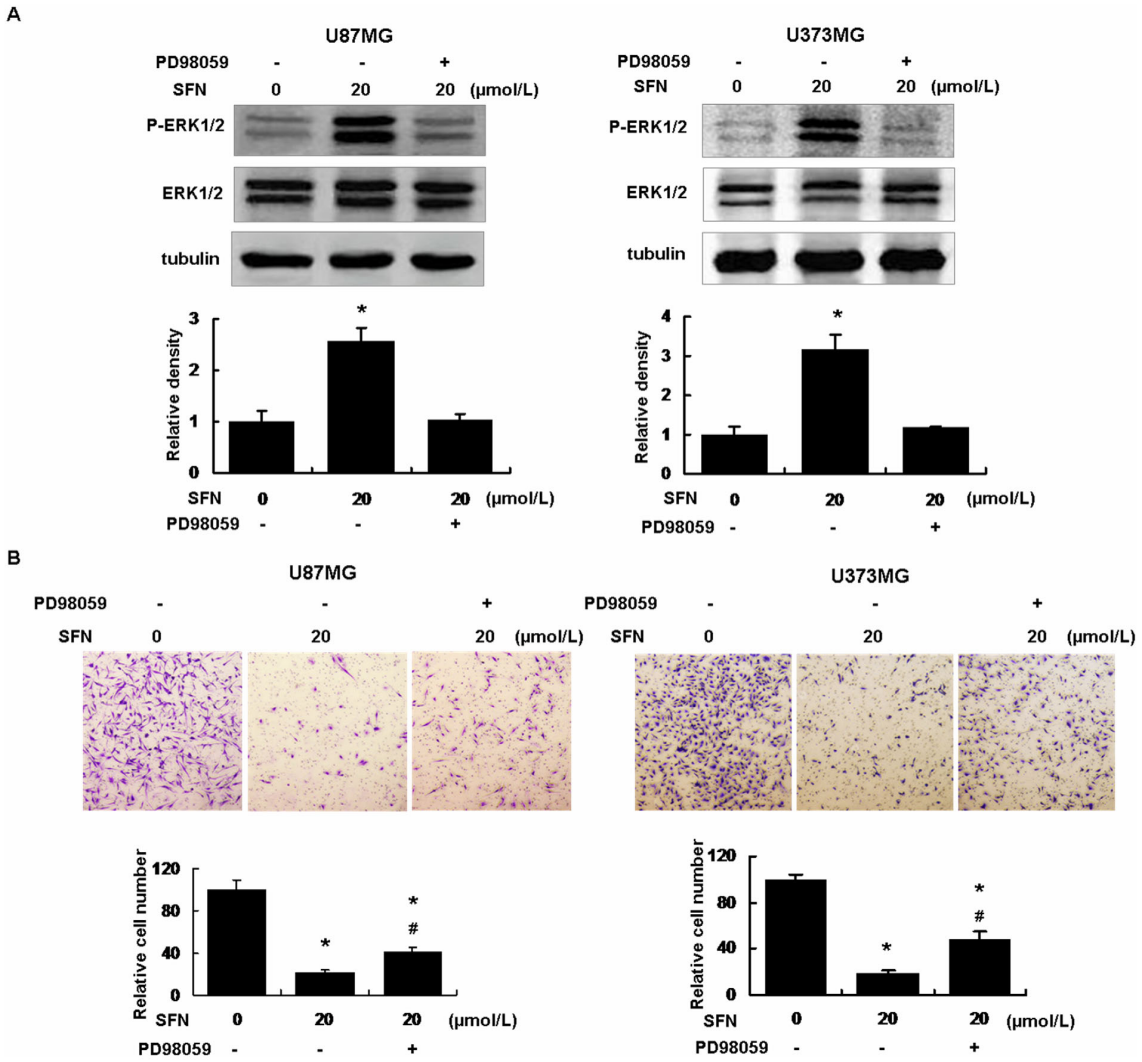
SFN Inhibited invasion via sustained activation of ERK1/2. (A) The U87MG and U373MG cells were respectively treated with SFN (20 µM) without or with PD98059 (25 µM) for 24 h, western blot showed that SFN activated ERK1/2 significantly. PD98059 decreased ERK1/2 phosphorylation. *, indicates *P<0.05* versus the control group. Data were shown as means ± SD from three separate tests. (B) We seeded 1×10^5^ cells in a 24-well plate with cell inserts, the cells were added with SFN (20 µM) without or with PD98059 (25 µM) for 24 h to detect cell invasion. Results showed that SFN inhibited cell invasion significantly versus control. SFN plus PD98059 reduced cell invasion inhibition compared with SFN-only. All procedures were performed as described in Methods. *, indicates *P<0.05* versus control, # indicates *P<0.05* versus SFN-only group. Data were shown as means ± SD from three separate tests.

Three different groups, including the control group (DMSO only), the SFN (20 µM) treatment, the SFN (20 µM) plus PD98059 (25 µM) treatment were made. After the cells were treated with PD98059 (25 µM), a specific ERK1/2 blocker, ERK1/2 phosphorylation was significantly diminished (Figure 6A). When the cells were treated with SFN (20 µM) alone, the cell invasion ability was decreased versus the control group (Figure 6B). After the cells were pretreated with PD98059 for 30 min, then treated with SFN for 24 h, the cell invasion ability in this group was increased significantly in contrast with the SFN only group. These results indicated that SFN inhibited invasion via sustained ERK1/2 activation in U87MG and U373MG cells.

### SFN Inhibited MMP-2 Expression and Activity via Sustained Activation of ERK1/2

**Figure 7:**
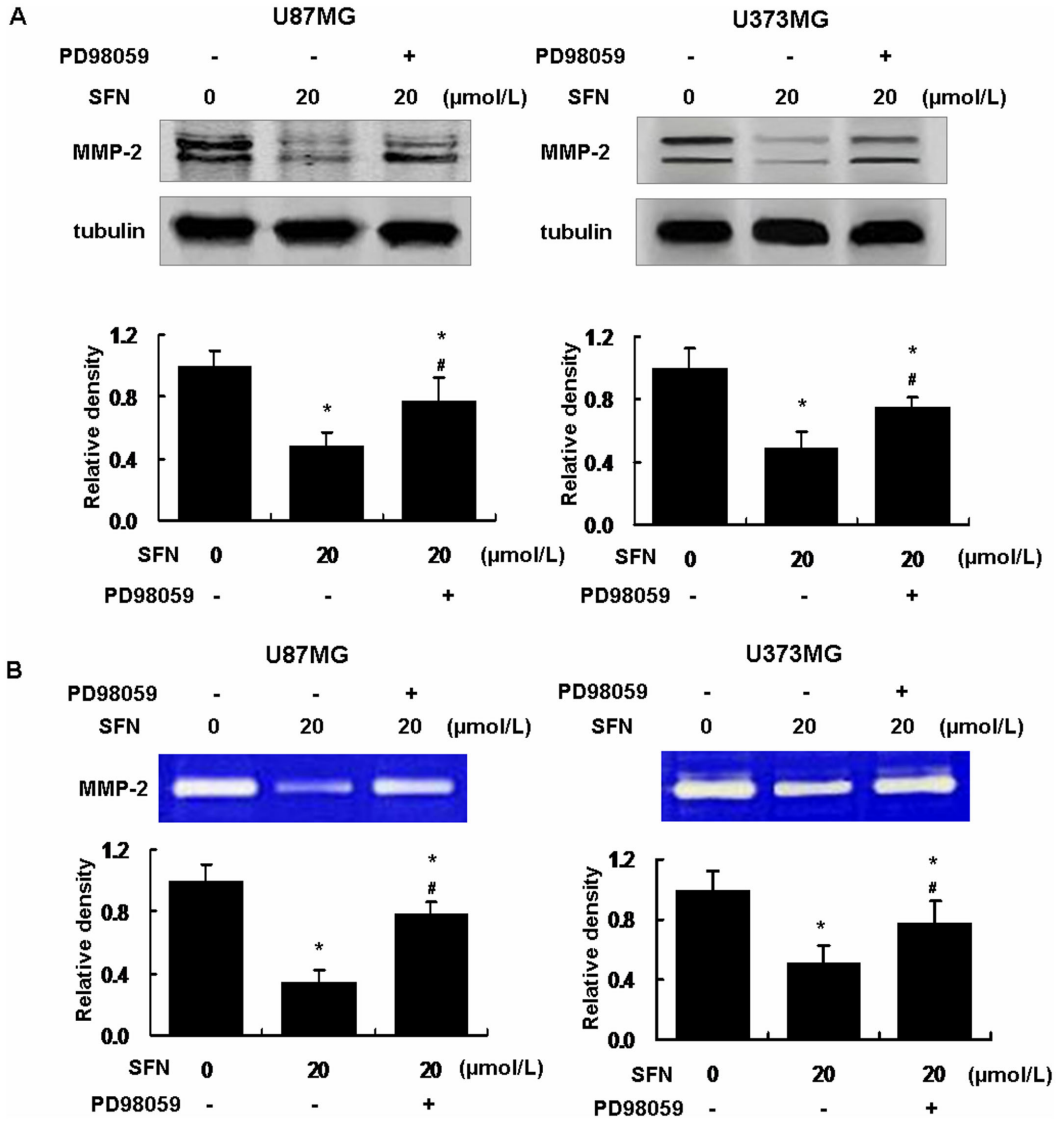
SFN decreased MMP-2 expression and activity in U87MG and U373MG cells. (A) SFN decreased MMP-2 expression via activated ERK1/2. We treated the cells with SFN (20 µM) without or with PD98059 (25 µM) for 24 h. Western blot showed that SFN significantly downregulated MMP-2 expression. SFN plus PD98059 reduced the downregulation of MMP-2 expression. The results indicated that SFN regulated MMP-2 expression via ERK1/2 activation. *, indicates *P<0.05* versus control. #, indicates *P<0.05* versus the SFN group. Data were shown as means ± SD from three separate tests. (B) SFN decreased MMP-2 activity via activated ERK1/2. We collected the conditioned medium from the above treatment for gelatin zymography assay as the methods. The results showed that SFN significantly decreased MMP-2 activity. SFN plus PD98059 significantly reduced MMP-2 activity inhibition versus the SFN-only. *, indicates *P<0.05* versus control. #, indicates *P<0.05* versus the SFN-only group. Data were shown as means ± SD from three separate tests.

To verify the molecular signaling for SFN-triggered invasion inhibition, we analyzed downstream MMP-2 expression. Western blot showed that SFN downregulated MMP-2 expression, and the level of MMP-2 expression was decreased to 0.48±0.09_SD_ (U87MG) and 0.49±0.10_SD_ (U373MG) when the cells were treated with 20 µM SFN for 24 h (control was set as 1) (Figure 7A). To determine whether decreased MMP-2 resulted from sustained phosphorylation of ERK1/2, the cells were treated with PD98059 (25 µM) combined with SFN (20 µM). Treatment with PD98059 and SFN dramatically reduced the downregulation of MMP-2 expression versus SFN-only treatment (Figure 7A). The results indicated that SFN downregulated MMP-2 expression via activating ERK1/2 in a sustained way. In addition, Gelatin zymography assay showed that MMP-2 activity was reduced significantly by SFN (20 µM); meanwhile, SFN (20 µM) downregulated MMP-2 activity via activating ERK1/2 in a sustained manner (Figure 7B).

### SFN Upregulated CD44v6 via Activating ERK1/2 in a Sustained Way

**Figure 8:**
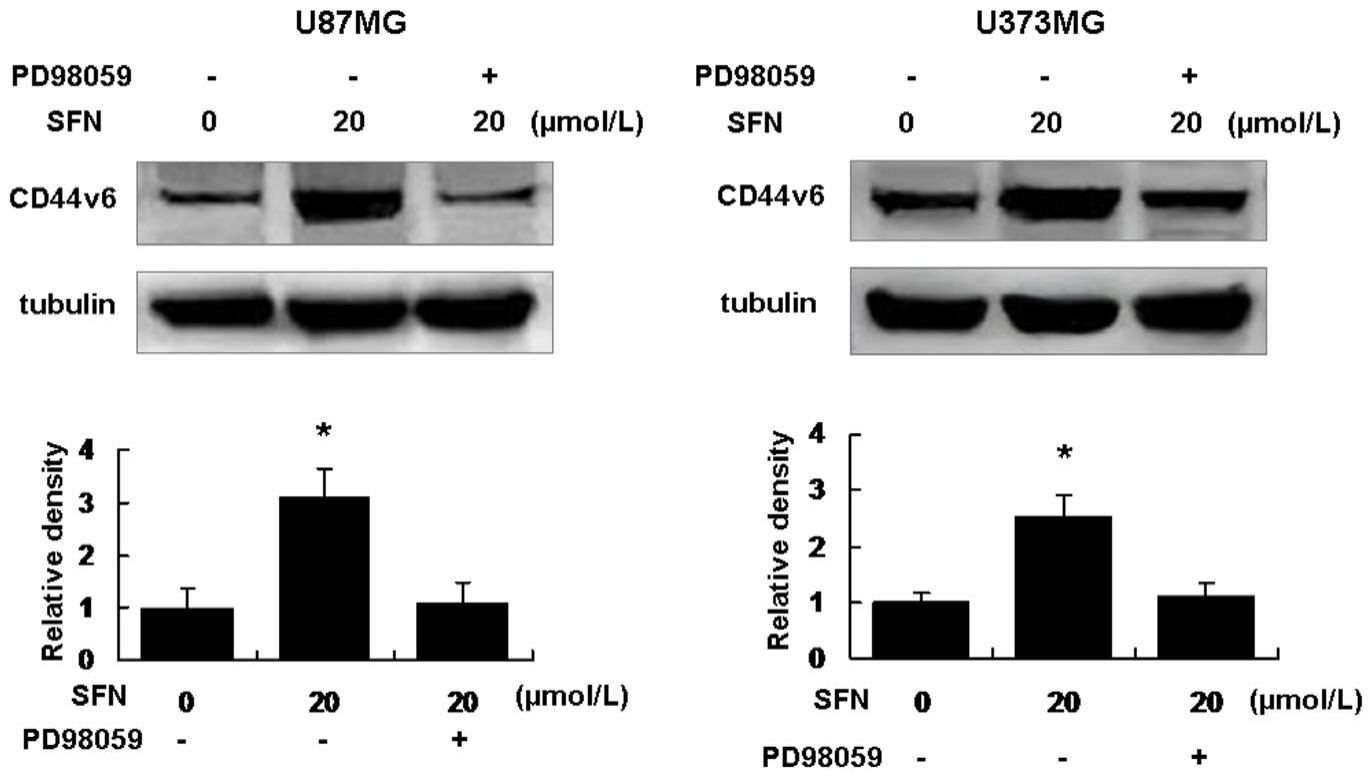
SFN upregulated CD44v6 expression in U87MG and U373MG cells. We treated the cells with SFN (20 µM) without or with PD98059 (25 µM) for 24 h. Western blot showed that SFN significantly upregulated CD44v6 expression. After PD98059 and SFN were added into the medium, CD44v6 expression was reduced significantly versus SFN-only group. That indicated SFN regulated CD44v6 expression via sustained ERK1/2 activation. *, indicates *P<0.05* versus control. Data were shown as means ± SD from three separate tests.

Western blot showed that SFN (20 µM) upregulated CD44v6, the level of CD44v6 expression was increased to 3.12±0.51_SD_ (U87MG) and 2.51±0.40_SD_ (U373MG) when the cells were treated with 20 µM SFN (control was set as 1) for 24 h (Figure 8). Interestingly, PD98059 (25 µM) significantly reduced the upregulation of CD44v6 versus SFN-only group (Figure 8). These indicated that SFN upregulated CD44v6 via sustained activation of ERK1/2 in the U87MG and U373MG cells.

## Discussion

Glioblastoma has highly invasive and fatal features. Tumor cells aggressively invade the surrounding tissue through infiltrating the brain parenchyma, resulting in the failure to clean up the tumor tissues by surgery and the recurrence of glioblastoma after surgery and radiotherapy. The sufferers have bad outcome with a median survival of only 15 months after multiple therapies. Hence there is an urgent need to develop a novel therapy to resist the tumor progress. Chemotherapy is a common strategy to treat glioblastoma in addition to surgery, radiotherapy and gene therapy. The first line agents, such as TMZ, have some defects in tumor resistance and cell toxicity. It is essential to find novel anti-tumor agent. SFN, as a food component, has no cell toxicity and tumor resistance, but has powerful anti-tumor properties. Studies demonstrated the SFN induced apoptosis in both T98G and U87MG glioblastoma cells through the activation of multiple molecular mechanisms [37]. In various tumor cells, SFN regulates many tumor-related events, including cell death, cell cycle and angiogenesis, but SFN-modulated tumor migration or invasion was only reported in some cancers [9], [10], [11], [12].

Here we determined that SFN inhibited tumor migration and invasion. Moreover, we found that SFN inhibited invasion via sustained activation of ERK1/2 and regulation of downstream invasion-related markers, MMP-2 and CD44v6. These results, on one hand, will provide supportive evidence for clinical use of SFN, on the other hand, will be of a great help to find more invasion mechanisms. Here we first demonstrated that SFN has anti-invasion potential via the specific signaling pathways in human glioblastoma U87MG and U373MG cells.

ERK1/2 signaling pathway is believed to play an important role in cancer chemotherapy due to its involvement in tumor cell proliferation, induced S-phase arrest, apoptotic cell death and differentiation. Our previous results showed that transient ERK1/2 activation had positive correlation to the expression and activity of downstream invasion-related protein MMP-2 in human glioblastoma U87MG cells [27], [28]. Studies showed that SFN caused a significant elevation in the phosphorylation of ERK1/2 [38]. Thus, we supposed that SFN triggered the ERK1/2 signaling pathway to regulate glioblastoma invasion. In our study, we detected the expression and activity of MMP-2 with the treatment of SFN, and used ERK1/2 inhibitor PD98059 to verify the ERK1/2 signaling pathway. The results showed that SFN inhibited invasion in U87MG and U373MG glioma cells via sustained ERK1/2 activation. The duration of ERK1/2 phosphorylation, as well as the duration of the accumulation of ERK1/2 in the nucleus is determined, at least in part, by nuclear phosphatases, which dephosphorylate ERK1/2 and impel them back to the cytoplasm [39]. Maybe SFN can induce phosphatases inhibitors to prevent the dephosphorylation of ERK1/2, and then the phospho-ERK1/2 gather in the nucleus, in other words, the activated ERK1/2 accumulated. Further study is required to verify the hypothesis. Sustained activation of ERK1/2 further decreased the expression and activity of MMP-2 resulting in the degradation of the ECM and basement membrane, as well as invasion inhibition. To our knowledge, no study proved that sustained activation of ERK1/2 led to invasion inhibition so far.

Previous studies demonstrated that ERK1/2 signaling regulated a number of transcription factors, such as activator protein-1 (AP-1) and nuclear factor-κB (NF-κB) [40]. And other studies showed that AP-1 and NF-κB acted independently or coordinately to regulate many genes involved in the regulation of MMP-2 and CD44v6 expression [41], [42]. Our results are consistent with the studies. Moreover, our finding enriches the ERK1/2 signaling pathway.

Changes in cell shape and plasticity in cytoskeletal dynamics are critically involved in cell adhesion, migration, invasion and the whole process of metastasis [43], [44]. SFN reduced the formation of pseudopodia and made morphological changes in U87MG and U373MG cells. Maybe that is one of the reasons why SFN inhibited cell migration and invasion in U87MG and U373MG cells. Studies showed that CD44 was related to the pseudopodia formation [45], [46], and CD44 participated in many important cellular processes, including adhesion and motility [47]. CD44v6 is an important member of the cell adhesion molecule CD44 family, and plays a crucial role in tumor invasion [48]. Little cell surface expression of CD44v6 was observed in samples of melanoma, neuroblastoma, glioma, cutaneous lymphomas and prostate cancers [46], [49]. Studies showed that over-expression of CD44v6 in a rat prostate cancer line decreased metastasis, and decreased expression of CD44v6 could be a predictor of poor prognosis in clinically localized prostate cancer [50]. These studies are consistent with our results that SFN upregulated CD44v6 and inhibited migration and invasion in U87MG and U373MG cells. Therefore, CD44v6 is an important target molecule of SFN in glioblastoma cells migration and invasion progress. Although SFN regulated CD44v6 via sustained ERK1/2 activation, we do not know how CD44v6 works for the migration and invasion inhibition yet.

Absorption and bioavailability are important factors when a compound is to be administered to humans. SFN could achieve high absolute bioavailability even at low dietary doses in rats [51]. In addition, studies reported that normal cells are more resistant to apoptosis induction by SFN than cancer cells [52], [53]. In other words, normal cells are less prone to the toxicity of SFN. This might imply that we can find an optimal dose of SFN to inhibit glioblastoma cells in vivo but safe for normal cells.

Clinically, we have to consider the fact that cancer patients already frequently harbor disseminated tumor cells in their blood, distant organs, and bone marrow when diagnosed [43]. Therefore, effective anti-metastatic therapeutics must be able to inhibit the proliferation and survival of already-disseminated cancer cells, rather than merely attempt to block escape of these cells from primary tumors. Fortunately, SFN not only inhibited migration and invasion, but also impaired the proliferation and survival in U87MG and U373MG cells in a dose-dependent manner. Thus, SFN is just the effective agent we desired.

In conclusion, here we revealed that SFN activated ERK1/2 in a sustained way, further downregulated MMP-2 and upregulated CD44v6, resulting in the inhibition of invasion in glioblastoma cells. Our results may give new insights into the molecular mechanisms of SFN regulation, and provide a potential way to treat glioblastoma.
